# Construction of a Set of Novel Transposon Vectors for Efficient Silencing of Protein and lncRNA Genes via CRISPR Interference

**DOI:** 10.1007/s12033-023-00675-5

**Published:** 2023-01-28

**Authors:** Maria Czarnek, Jakub Kochan, Mateusz Wawro, Rafał Myrczek, Joanna Bereta

**Affiliations:** Department of Cell Biochemistry, Faculty of Biochemistry, Biophysics and Biotechnology, Jagiellonian University in Kraków, Gronostajowa 7, 30-387 Kraków, Poland

**Keywords:** CRISPR interference, Sleeping beauty transposon, Constitutive transgene expression, Inducible transgene expression, Mouse ADAM10, *NORAD*

## Abstract

In recent years, CRISPR interference (CRISPRi) technology of gene silencing has emerged as a promising alternative to RNA interference (RNAi) surpassing the latter in terms of efficiency and accuracy. Here, we describe the construction of a set of transposon vectors suitable for constitutive or tetracycline (doxycycline)-inducible silencing of genes of interest via CRISPRi method and conferring three different antibiotic resistances, using vectors available via Addgene repository. We have analyzed the performance of the new vectors in the silencing of mouse *Adam10* and human lncRNA, *NORAD.* The empty vector variants can be used to efficiently silence any genes of interest.

## Introduction

Over the years, RNA interference (RNAi) was one of the most influential technics applied to study gene functions despite quickly acquired awareness of its serious shortcomings [1]. The attempts to improve this technology, made in the absence of alternatives, did not eliminate the disadvantages resulting from the very essence of natural RNAi phenomenon. The major drawbacks of RNAi technology harnessing designed siRNA or shRNA molecules, which mimic miRNA or pre-miRNA molecules, include frequent off-target effects, possible cytotoxicity, and interference with miRNA pathways [2–6]. We also have encountered very unfavorable effects of RNAi technology in our research. While studying the impact of shRNA-mediated silencing of expression of ADAM (A Disintegrin and Metalloproteinase) family members, we found that some shRNAs targeting *Adam17* affected also the expression of *Adam10* and a number of *Adam* targeting shRNAs revealed that anti-inflammatory activity did not correlate with gene silencing [7]. What is more, we demonstrated that a control, non-targeting shRNA for MISSION® shRNA library exerts potent off-target effects. It silences *SNRPD3*, which strongly affects cell viability and thus may lead to misinterpretation of RNAi results [8]. In recent years, serious pitfalls of RNAi-based analyses with far-reaching consequences have also been demonstrated by Lin et al. [9, 10].

The discovery of bacterial CRISPR-Cas9 (Clustered Regularly Interspaced Short Palindromic Repeats-CRISPR-associated protein 9) system and its applicability in eukaryotic cells resulted in the development of alternative methods of gene silencing [11–13]. Initially, CRISPR-Cas9 was used for genome editing, a laborious, long-term procedure, which is not completely free from off-target effects resulting from DNA breaks randomly introduced by Cas9 enzyme [3]. Almost a decade ago another technic based on the CRISPR-Cas9 system, namely, CRISPR interference (CRISPRi) was developed, in which enzymatically inactive Cas9 (dead Cas9, dCas9) targeted to a gene of interest by a properly designed single guide RNA (sgRNA) inhibits the binding of RNA polymerase to the gene promoter or blocks the progress of transcription, thus effectively reducing the level of its expression [14]. The method has been constantly improved via, among others, optimizing selection of best sgRNA sequences [15–18] and generating fusion proteins in which dCas9 is complexed with known transcriptional repressors [19–21]. Although not completely flawless, CRISPRi outcompetes RNAi in terms of reducing off-target effect [22–24].

Here, we describe the design of a novel CRISPRi vector, which we constructed using non-commercial Addgene-deposited plasmids. We generated the vector variants that knockdown mouse *Adam10* expression constitutively or upon induction with doxycycline with three different antibiotic resistances. Their empty counterparts can be used to target the expression of any gene of interest upon simple insertion of an appropriate gene targeting sequence into sgRNA backbone. Both empty vectors as well as these targeting *Adam10* and *NORAD* (Non-coding RNA Activated by DNA Damage) are available via Addgene repository (plasmid numbers for empty vectors: 196074, 196076, 196078, 196080, 196082, 196084, for vectors targeting *Adam10*: 196075, 196077, 196079, 196081, 196083, 196085, for the vector targeting *NORAD*: 196086).

## Materials and Methods

### Construction of CRISPRi Vectors for Constitutive Expression of Cas9-KRAB-MeCP2 Fusion Protein and sgRNA

dCas9-KRAB-MeCP2 repressor was amplified from dCas9-KRAB-MeCP2 [21] (a gift from Alejandro Chavez & George Church; Addgene plasmid #110821). hU6 promoter and sgRNA scaffold containing SapI sites were amplified from pX330 containing SapI sites instead of BbsI sites [8]. EF1α promoter and a fragment comprising most of the plasmid backbone (including AmpR, ori, PuroR) were amplified from pSBbi-Pur [25] (a gift from Eric Kowarz, Addgene plasmid # 60523). The DNA fragments were assembled using NEBuilder HiFi DNA Assembly Cloning Kit (New England Biolabs). The resulting vector was named pSBbi-Pur-dCas9-KRAB-MeCP2-hU6-SapI.

Three different sgRNA sequences targeting sites near the transcription start site of mouse *Adam10* gene were designed using GPP sgRNA Designer (presently CRISPick, Broad Institute) (Table 1). A single sgRNA sequence targeting *NORAD* comes from Elguindy et al. [26] (Table 1). The oligonucleotides containing compatible ends with the SapI-digested plasmid were annealed in Taq buffer and cloned into the vector in one restriction–ligation reaction using SapI and T4 DNA ligase. The resulting vectors were named pSBbi-Pur-dCas9-KRAB-MeCP2-hU6-A10 (variant 1, 2, and 3) or pSBbi-Pur-dCas9-KRAB-MeCP2-hU6-*NORAD*. The proper insertions of the gene targeting sequences were confirmed by sequencing (Genomed, Warsaw).

**Table 1:** Sequences of used oligonucleotides

Gene of interest	Oligonucleotide sequences*
Gene targeting sequences	
* Adam10_1*	T: **ACC**GCGAGAGGGAGGCGCTTCGCC** B: **AAC**GGCGAAGCGCCTCCCTCTCGC
* Adam10_2*	T: **ACC**GGCTCGTCGGGACCCAGCGC B: **AAC**GCGCTGGGTCCCGACGAGCC
* Adam10_3*	T: **ACC**GACCGCGGTTAACCCGTGAGG B: **AAC**CCTCACGGGTTAACCGCGGTC
* NORAD*	T: **ACC**GTTCTCTGCGCTGGCAAGAG B: **AAC**CTCTTGCCAGCGCAGAGAAC
Primers	
* Adam10*	F: CCGGGCTCTCCATGTAATGA R: CCAGTGAGCCACAATCCAC
* Adam17*	F: AGGGTTCTAGCCCACATAGGA R: TGGAGACTGCAAACGTGAAA
* Oas1*	F: AGGGCCTCTAAAGGGGTCAA R: ACCTCGCACAGCTGTTTCTT
* Ifit1*	F: GCTCTGCTGAAAACCCAGAGA R: AAGGAACTGGACCTGCTCTGA
* Eef2*	F: CCACGGCAAGTCCACGCTGAC R: AGAAGAGGGAGATGGCGGTGGATT
* Polr2b*	F: GGATTCTGGGAACGTCGGAG R: CCGGAGTGATCTCATCGTCG
* NORAD*	F: TGATAGGATACATCTTGGACATGGA R: TGGACACATCTGCATACATCTCT
* EEF2*	F: GAGAGCATATCATCGCGGGC R: AGAGCACGTTCGACTCTTCA

*T* top, *B* bottom, *F* forward, *R* reverse

^*^All oligonucleotides were provided by Genomed, Warsaw

^**^Underlined—gRNA, Bold—complementary to SapI overhangs

To obtain CRISPRi vectors targeting *Adam10* with blasticidin S or hygromycin B resistances, we cloned the most efficient sgRNA sequence into pSBbi-Bsd-dCas9-KRAB-MeCP2-hU6-SapI and pSBbi-Hyg-dCas9-KRAB-MeCP2-hU6-SapI generated as described above using pSBbi-Bla or pSBbi-Hyg scaffolds [25] (gifts from Eric Kowarz, Addgene plasmids # 60526 and # 60524). The proper insertion of *Adam10* targeting sequence was confirmed by sequencing (Genomed, Warsaw).

### Construction of Inducible CRISPRi Vectors

Inducible CRISPRi vector conferring puromycin resistance (pSBtet-Pur-dCas9-KRAB-MeCP2-hU6-SapI) was described previously [8]. CRISPRi vectors conferring blasticidin S and hygromycin B resistance (*BsdR* and *HygR,* respectively) were prepared as follows: pSBtet-Pur-dCas9-KRAB-MeCP2-hU6-SapI was digested with BsrGI and DraIII; the fragment containing a part of RPBSA promoter, rtTA and P2A were PCR-amplified from pSBtet-Pur-dCas9-KRAB-MeCP2-hU6-SapI; *BsdR* and *HygR* genes were PCR-amplified from pSBbi-Bla and pSBbi-Hyg, respectively. DNA fragments were assembled using NEBuilder HiFi DNA Assembly Cloning Kit (New England Biolabs). The resulting vectors were named pSBtet-Bla-dCas9-KRAB-MeCP2-hU6-SapI and pSBtet-Hyg-dCas9-KRAB-MeCP2-hU6-SapI. The *Adam10* targeting sequence was cloned into SapI sites as described above. The proper insertion of resistance gene- and *Adam10* targeting sequences were confirmed by sequencing (Genomed, Warsaw).

### Cell Culture and Transfection

Mouse colon carcinoma MC38CEA cell line [8] and HeLa (human cervical adenocarcinoma, ATCC CCL-2) were cultured in DMEM (Biowest) supplemented with 10% heat-inactivated fetal bovine serum (FBS) or with tetracycline-negative FBS (Capricorn Scientific, Ebsdorfergrund, Germany) under standard conditions. The cell cultures were tested by PCR for mycoplasma contamination using mycoplasma rDNA-specific primers and carried out without Pen/Strep to avoid unnoticed mycoplasma contamination.

The cells were grown in 6-well plates and when reached 80–90% confluency were transfected with 950 ng of a respective CRISPRi plasmid together with 50 ng of pCMV(CAT)T7-SB100 vector encoding SB100X transposase (a gift from Zsuzsanna Izsvak; Addgene plasmid # 34879)[27] using jetPRIME (Polyplus Transfection) for MC38CEA or Lipofectamine 3000 (Thermo Fisher Scientific) for HeLa cells. One day after transfection an appropriate selection antibiotic was added to the cell cultures at following concentrations: puromycin—5 µg/ml for MC38CEA and 1 µg/ml for HeLa cells, blasticidin S—3 or 5 µg/ml, or hygromycin B—300 or 500 µg/ml. The cells were cultured in the presence of a selection antibiotic for at least a week.

### RT-qPCR

After antibiotic selection, the cells were grown in 12-well plates. The cells transfected with the vectors with doxycycline-inducible expression of dCas9-based repressor were cultured for 3 days in the absence (control) or presence of doxycycline (added daily to a concentration of 100 ng/ml) prior to RNA isolation. RNA was isolated using Fenozol (A&A Biotechnology) and, after removal of DNA by TURBO DNase (Thermo Fisher Scientific), purified with Clean Up RNA Concentrator columns (A&A Biotechnology). Equal amounts of RNA (1 μg) were subjected to reverse transcription using M-MLV polymerase (Promega) and a mixture of oligo(dT)15 (Genomed) and random hexamer primers (Promega). The levels of gene expression were evaluated via qPCR on an Eco Real-Time PCR System (Illumina) using AceQ qPCR SYBR GreenMix (Vazyme Biotech) and specific primers listed in Table 1. The fold changes in gene expression were quantified using ΔΔCt method in relation to the expression of two reference genes *Eef2* and *Polr2b* for mouse cells and *EEF2* for human cells.

### Western Blotting

The cells were lysed in ice-cold RIPA buffer enriched with Halt Protease Inhibitor Cocktail containing 5 mM EDTA (Thermo Scientific). Protein samples (20 μg) were subjected to tris-glycine SDS-PAGE containing trichloroethanol, which enables UV-induced protein visualization in the gel [28]. The proteins were transferred onto 0.45 μm PVDF membrane (Immobilon FL, Merck) and probed with rabbit anti-ADAM10 (ab1997, Abcam) at 1:5000 and then with HRP-conjugated secondary antibody (anti-rabbit, Cell Signaling Technology, at 1:10,000). Bands were developed with Immobilon Western Chemiluminescent HRP Substrate (Merck) and visualized using Fusion FX (Vilber Lourmat).

### Fluorescence Microscopy—Single Molecule RNA Fluorescence In Situ Hybridization (smRNA FISH)

Fluorescence imaging procedures were essentially performed as described previously [29]. Concisely, HeLa cells were plated on glass coverslips (#1.5H, Menzel Gläser) in 12-well culture plates at a density of 25,000 cells/well and cultured for 3 days in the absence (control) or presence of doxycycline (added daily to a concentration of 100 ng/ml). Next, the cells were washed twice in RNase-free PBS (Thermo Fisher Scientific) and fixed for 15 min in 4% methanol-free formaldehyde (Thermo Fisher Scientific) in RNase-free PBS at room temperature. Following fixation, specimens were washed three times in RNase-free PBS and smRNA FISH procedure was carried out according to the manufacturers’ instructions (Biosearch Technologies, *NORAD* probe blend (labeled with Quasar 570 dye), final concentration: 250 nM). Hybridizations were performed overnight in the dark at 37 °C in humidifying chamber. Finally, all samples were counterstained using DAPI (Thermo Scientific) and mounted onto slides in ProLong Glass Mounting Medium (Thermo Scientific). After overnight curing at room temperature, the prepared specimens were imaged using a Leica DMi6 B widefield fluorescence microscope (Leica Microsystems) equipped with a Leica DMC5400 camera (Leica Microsystems) and a 63×1.3 NA oil objective (Leica Microsystems). The following filter sets (Leica Microsystems) were used: A4 for detection of DAPI and RHOD ET for detection of Quasar 570 Dye. The images were analyzed and *NORAD* molecules were quantified after deconvolution of about 35 z-sections with 0.3 μm spacing for each sample using Huygens Software (Scientific Volume Imaging). Final image adjustments (for presentation purposes only) were performed using ImageJ 1.53q (National Institutes of Health) [30].

### Additional Information

Data analysis was performed using Microsoft Excel (Excel 2016) or GraphPad Prism v. 9 and all graphs were created using GraphPad Prism v. 9 (GraphPad Software) and CorelDRAW 2020 (Corel).

## Results and Discussion

The first goal of our work was to generate a single vector for stable expression of both elements of CRISPRi system: dCas9-based repressor and sgRNA, using non-commercial resources available via Addgene repository. As a transcriptional repressor, we chose dCas9-KRAB-MeCP2, in which dCas9 is fused to bipartite repressor domain containing Krüppel-associated box (KRAB) and methyl CpG binding protein 2 (MeCP2) domains, known to play roles in blocking transcription [31, 32]. The sequence contains two nuclear localization signals (NLS) directing dCas-based repressor to the nucleus. The vector coding for this fusion protein generated by Yeo et al. has been proved to efficiently inhibit expression of studied genes [21].

dCas9-KRAB-MeCP2 fusion protein alone is encoded by a sequence of more than 5300 bp and this size predestined the choice of a high-capacity transposon system for the construction of our vector. Transposons are natural, mobile genetic elements. They are flanked by terminal inverted repeat sequences (TIRs) recognized by transposase, an enzyme with excisase and integrase activities. The genetically modified transposons are used as efficient tools for stable incorporation of various DNA sequences into the genomes of target cell. A transposon system consists of two elements: a plasmid containing a transgene placed between TIR sequences and a compatible transposase delivered to the cells as a cDNA, mRNA, or protein. The activity of transposase empowers the efficiency of genomic integration comparable to that of viral vectors. Three transposon systems applicable for mammalian cells genetic modifications are currently available: *Sleeping Beauty*, *piggyBac*, and *Tol2* [33]. We have chosen *Sleeping Beauty* system, which was reconstructed from the dormant salmonid Tc1/mariner-type transposon via elimination of inactivating mutations [34]. The major advantage of this system is close-to-random distribution of integration sites, not biased towards transcriptionally active sites, as observed for other transposon and viral vectors [33, 35]. The comparison of SB characteristics with other commonly used vectors is presented in Table 2. As a scaffold for our vector, we used the version of SB plasmid redesigned by Kowartz et al. to simplify cloning procedure, introduce selection markers, and optimize promoters, enhancers, and polyA sequences [25].

**Table 2:** Transposon vector characteristics compared to other commonly used vectors

	Sleeping Beauty transposon vector	Lentiviral vector	Non-transposon plasmid
Safety	Safe	Requires special safety facility and qualified personnel	Safe
Efficiency of genomic insertion*	High, preferentially at TA dinucleotides (unbiased)	High, biased towards promoters and transcribed genes	Low
Time for final vector construction	Short	Long	Short
Time for cell line generation	Short	Short	Long
Cost	Depending on transfection method	High	Depending on transfection method

*for proliferating cells

**Fig. 1:**
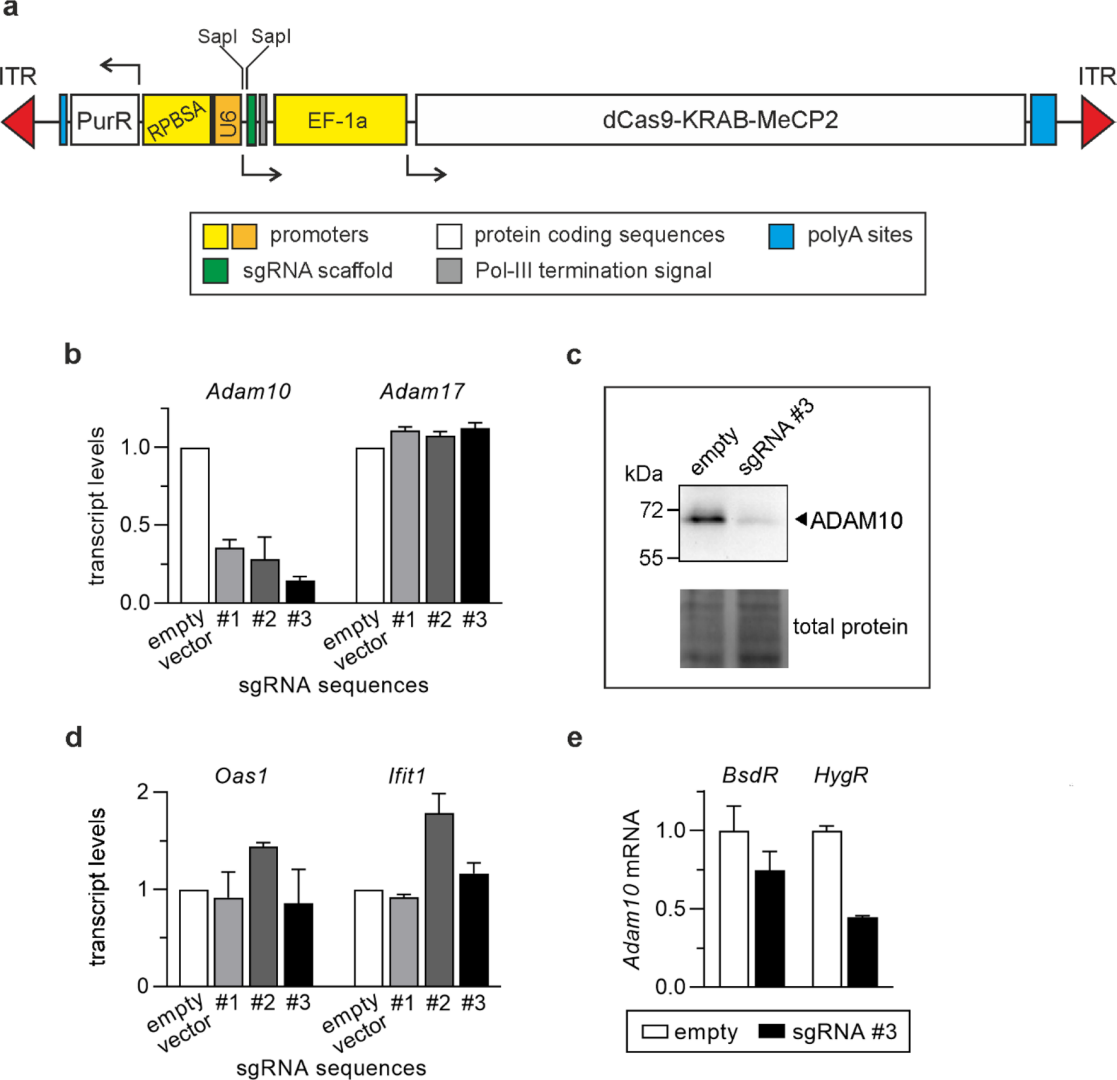
Analysis of functionality of CRISPRi vectors for constitutive silencing of *Adam10*. **a** the diagram of the fragment of pSBbi-Pur-dCas9-KRAB-MeCP2-hU6-SapI flanked by transposase recognition motifs (ITR). **b** RT-qPCR analysis of *Adam10* and *Adam17* levels in MC38CEA cells transfected with pSBbi-Pur-dCas9-KRAB-MeCP2-hU6-SapI variants encoding three different sgRNAs targeting *Adam10*. The relative levels of the transcripts in the cells transfected with the vector not containing gene targeting sequence were taken as 1 (white bars). **c** Western blot analysis of ADAM10 levels in MC38CEA transfected with pSBbi-Pur-dCas9-KRAB-MeCP2-hU6-SapI or pSBbi-Hyg-dCas9-KRAB-MeCP2-hU6-SapI encoding sgRNA sequence #3 targeting *Adam10.* Total protein loading is presented in the bottom panel. **d** RT-qPCR analysis of interferon-inducible transcripts in MC38CEA cells transfected as in **a**. **e** RT-qPCR analysis of *Adam10* levels in MC38CEA cells transfected with pSBbi-Bsd-dCas9-KRAB-MeCP2-hU6-SapI or pSBbi-Hyg-dCas9-KRAB-MeCP2-hU6-SapI encoding sgRNA sequence #3 targeting *Adam10*. The relative level of *Adam10* in non-transfected wild type (WT) cells was taken as 1. **b, d, e**. Data are shown as mean values from 3 independent experiments for *Adam10* and two for *Adam17*, *Oas1* and *Ifit1*. Error bars represent standard deviation (SD). **c** Representative image of two independent experiments is shown

The fragment directing sgRNA expression was derived from pX330-U6-Chimeric_BB-CBh-hSpCas9 (a gift from Feng Zhang; Addgene plasmid # 42230), which contains a sequence coding for a constant fragment of sgRNA required for recognition of sgRNA by dCas9 under U6 promoter and transcription termination signal for RNA polymerase III [11]. In the original plasmid, the site for the cloning of a gene targeting sequence into the sgRNA backbone was created by two sequences recognized by BbsI, a type IIS restriction enzyme, which generates non-palindromic overhangs ensuring proper cloning orientation. We replaced BbsI sites with the ones recognized by SapI, another type IIS enzyme, to prevent cleavage of dCas9-KRAB-MeCP2-coding sequence containing four BbsI sites [8]. The amplified DNA fragments (the scaffold, dCas9-KRAB-MeCP2, and U6 promoter together with sgRNA-encoding cassette) were assembled into pSBbi-Pur-dCas9-KRAB-MeCP2-hU6-SapI. The diagram of the fragment incorporated into DNA of transfected cells is presented in Fig. 1a.

We next cloned three different RNA sequences targeting mouse *Adam10* into SapI sites. They were chosen from the sequences designated by GPP sgRNA Designer, presently CRISPick (Broad Institute) [15, 18]. We selected the sequences with presumed very low off-target activity. The sequences are located in the first exon and comprise nucleotides: (1) 85–105, (2) 124–143, and (3) 171–190 from the transcription start site (TSS) and are followed, respectively, by GGG, CGG, and AGG PAM motifs. According to Gilbert et al., the window of DNA regions efficiently targeted by dCas9-KRAB comprised fragments between − 50 and + 300 bp relative to TSS with a maximum of inhibitory activity observed for the + 50–100 bp region [24]. Even broader optimal DNA window was indicated for dCas9-KRAB-MeCP2-executed repression [21]. All three sequences substantially inhibited expression of *Adam10* in MC38CEA cells transfected with pSBbi-Pur-dCas9-KRAB-MeCP2-hU6-SapI_A10 variants and SB100X transposase-encoding vector (Fig. 1b). Their specificity towards *Adam10* was indicated by the lack of impact on the expression of *Adam17* (the closest relative of *Adam10)* (Fig. 1b). The decrease in *Adam10* mRNA levels was accompanied by diminished levels of ADAM10 protein (Fig. 1c), which confirms our previous observations of a correlation between silencing of mouse *Adam10* at the transcript and protein levels [7].

The expression of neither sgRNA induced the so-called interferon response since the expression of two interferon-inducible genes, *Oas1* and *Ifit1* were not significantly stimulated in the transfected cells (Fig. 1d). The strongest inhibition of *Adam10* expression was observed for the vector bearing gene targeting sequence #3. Initially, when lone dCas9 was used as a repressor in CRISPRi, the use of multiple sgRNAs potentiated gene expression knockdown [14]. However, if the repressor was dCas9-KRAB-MeCP2, the use of multiple sgRNAs did not silence gene expression more than a single, most potent sgRNA [21]. Therefore, the sequence #3 was chosen for all subsequent variants of CRISPRi vectors targeting *Adam10*.

We prepared two additional vectors constitutively targeting *Adam10* and providing resistance to blasticidin S (*BsdR*) or hygromycin B (*HygR*) following the procedure described above and using pSB scaffolds with different antibiotic resistances. However, these vectors showed lower effectiveness in *Adam10* silencing than the one conferring puromycin resistance (Fig. 1e). The low efficiency of the vector providing resistance to blasticidin S is in agreement with the recently observed correlation between type of selectable marker and the levels of expression of recombinant proteins [36]. The authors showed that the expression of genes of interest may be significantly lower when blasticidin S or G418 are applied as selection antibiotics instead of other commonly used such as zeocin, puromycin, or hygromycin. It is possible that in MC38CEA cells transfected with *BsdR-*expressing vector the levels of dCas9-KRAB-MeCP2 were too low to efficiently block *Adam10* transcription.

**Fig. 2:**
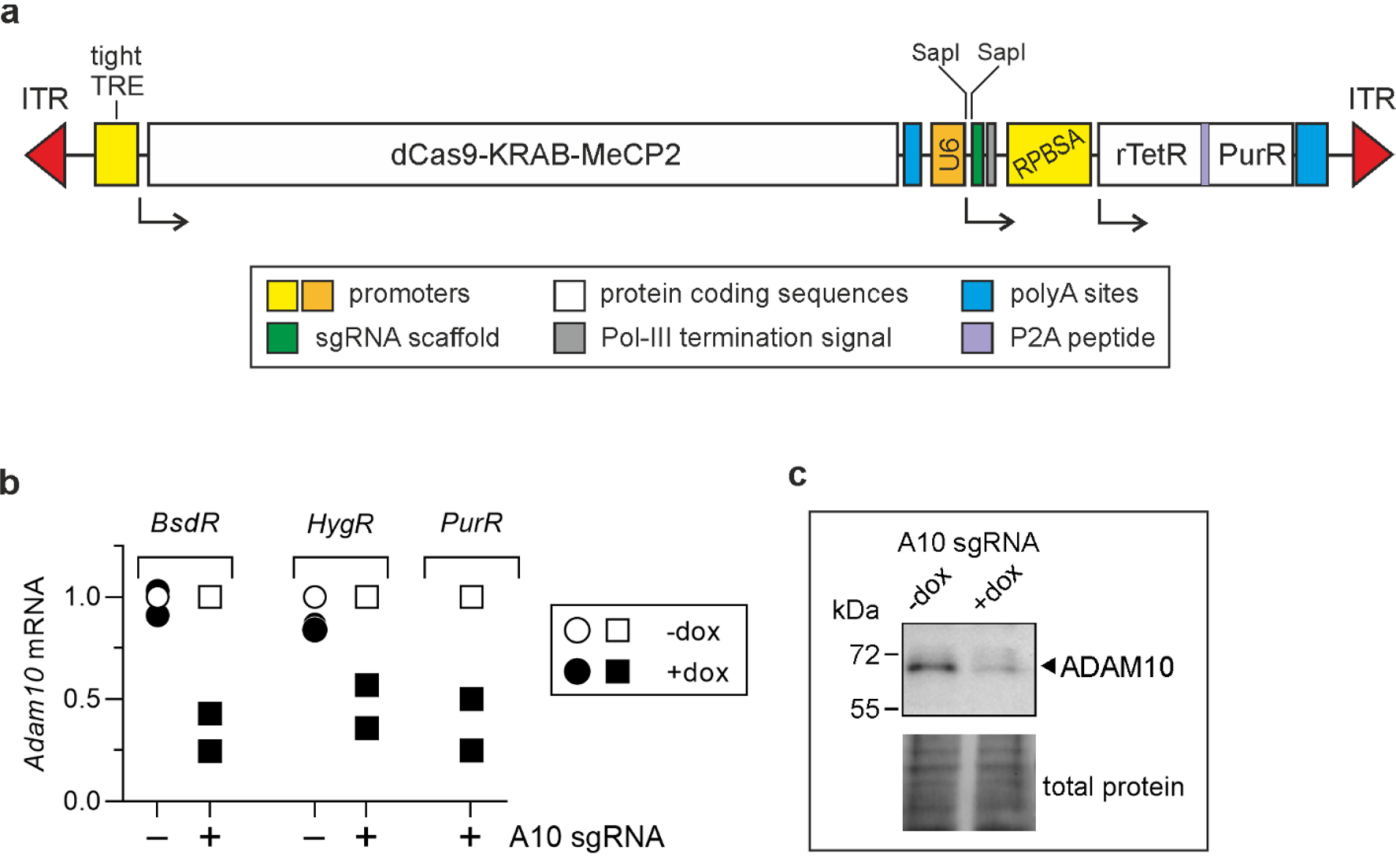
Analysis of functionality of CRISPRi vectors for inducible silencing of *Adam10*. **a** the diagram of the fragment of pSBtet-Pur-dCas9-KRAB-MeCP2-hU6-SapI flanked by transposase recognition motifs (ITR). **b** RT-qPCR analysis of *Adam10* transcript levels in MC38CEA cells transfected with the vectors conferring distinct antibiotic resistances and not containing (circles) or containing sgRNA targeting *Adam10* (squares) cultured for 72 h in the absence or presence of doxycycline. The relative levels of *Adam10* in the cells that were not treated with doxycycline were taken as 1 (white symbols). The lack of influence of the empty vector conferring puromycin resistance was documented previously [8]. **c** Western blot analysis of ADAM10 levels in MC38CEA transfected with pSBtet-Pur-dCas9-KRAB-MeCP2_hU6-A10 and cultured for 72 h in the absence or presence of doxycycline*.* Even protein loading is presented in the bottom panel. Representative image of two independent experiments is shown

To create doxycycline-inducible set of CRISPRi vectors targeting *Adam10*, we have used previously generated vector for doxycycline-inducible expression of genes of interest [8]. This vector with puromycin resistance (Fig. 2a) was used for generation of its *BsdR* and *HygR* variants. The *Adam10* targeting sequence was cloned into all of them, MC38CEA cells were transfected with the vectors and after antibiotic selection the expression of dCas9-KRAB-MeCP2 was induced with doxycycline. In two independent experiments we observed a moderate or substantial decrease of *Adam10* transcript levels (Fig. 2b) accompanied by diminished *ADAM10* protein levels as analyzed for the *PurR* vector variant (Fig. 2c). In the case of this set of vectors, *PurR* and *BsdR* variants were more efficient than the one conferring resistance to hygromycin. The increased concentrations of blasticidin S and hygromycin B during selection process did not improve effectiveness of doxycycline-induced *Adam10* silencing (data not shown).

We have also evaluated the applicability of our CRISPRi vectors, both with constitutive and inducible expression of dCas9-KRAB-MeCP2 to silence the expression of one of human long non-coding RNAs, namely *NORAD*. We have cloned the sequence encoding the oligonucleotide previously shown to efficiently target *NORAD* [26], into our CRISPRi vectors conferring puromycin resistance.

**Fig. 3:**
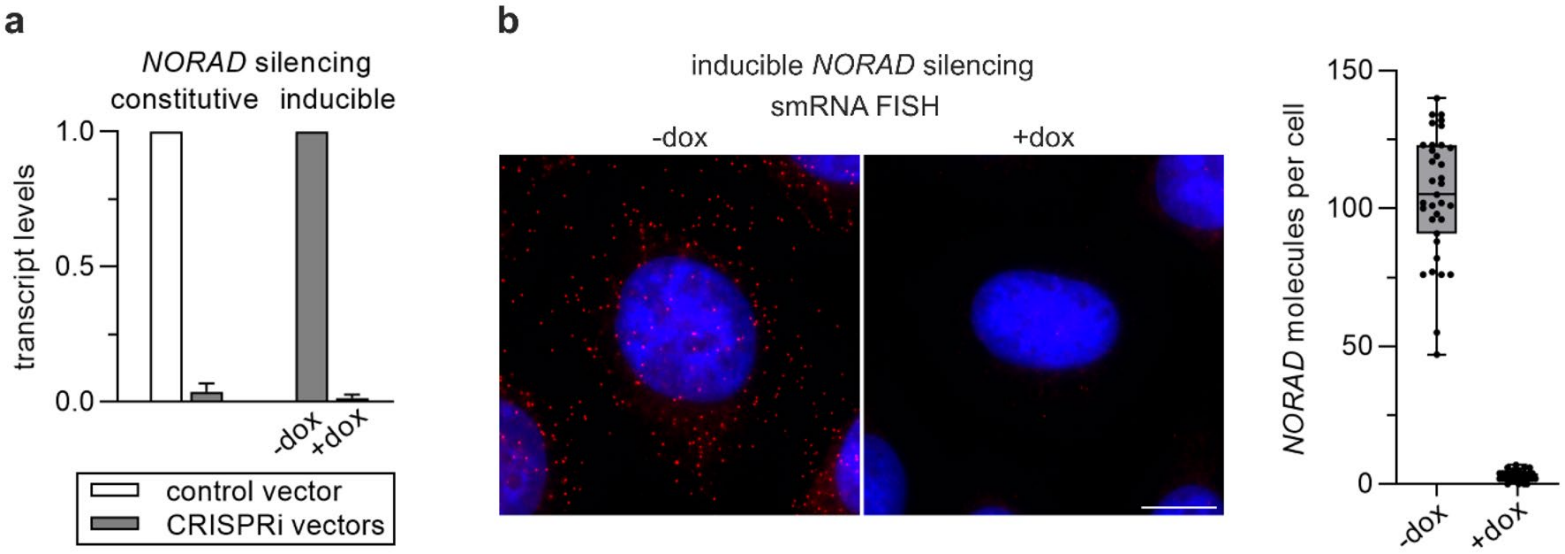
Analysis of effectiveness of constitutive and inducible silencing of *NORAD* expression using novel CRISPRi vectors. **a** RT-qPCR analysis of *NORAD* levels in HeLa cells transfected with CRISPRi vectors. In the case of the vector for inducible *NORAD* knockdown, the cells were incubated for 3 days in the absence or presence of doxycycline before the isolation of RNA. The relative levels of *NORAD* in HeLa cells transfected with the control vector for the constitutive silencing (not containing *NORAD* targeting sequence) or in HeLa cells transfected with CRISPRi vector for the inducible silencing and cultured without doxycycline were taken as 1. Data are shown as mean values from 3 independent experiments. Error bars represent standard deviation (SD). **b** smRNA FISH imaging of *NORAD* in HeLa cells transfected with CRISPRi vector for the inducible silencing and incubated for 3 days in the absence or presence of doxycycline. Left panel—exemplary fluorescence images of HeLa cells. *NORAD* molecules are stained with specific probes (red dots) and nuclei with DAPI (blue). Scale bar = 10 μm. Right panel—quantification of *NORAD* molecules from 35 z-sections randomly chosen for each sample. The points represent values obtained from individual cells; box limits—25th and 75th percentiles; box bar—median, whiskers—minimum to maximum range

The expression of *NORAD* in HeLa cells was evaluated by RT-qPCR and by counting *NORAD* molecules in the cells using smRNA FISH. The results were fully consistent and indicated that both vectors led to profound inhibition of *NORAD* expression in HeLa cells upon transfection (in the case of the vector for constitutive silencing, Fig. 3a) or upon transfection and switching on dCas9-KRAB-MeCP2 synthesis with doxycycline (in the case of the vector for inducible silencing, Fig. 3a, b).

The level of expression of this RNA in HeLa cells is high as the number of *NORAD* molecules exceeds a hundred (Fig. 3b) and the transcription of *NORAD* is driven by RNA polymerase II, as is the transcription of genes encoding proteins. The impressive, almost complete repression of *NORAD* transcription in HeLa cells supports our belief in excellence of the vectors we constructed to silence the expression of genes encoding both lncRNA and proteins. However, other factors such as selection of the best sgRNA sequence, efficiency of transfection of a given cell line, sensitivity of a given cell line to selection antibiotics may all influence the effectiveness of CRISPRi-mediated gene silencing, and thus every single experimental setting requires optimization for the satisfactory results. The summary of advantages and drawbacks of CRISPRi method in comparison to CRISPR-Cas9-mediated knockout (CRISPR ko) and to shRNA-mediated gene silencing (shRNAi) is presented in Table 3.

**Table 3:** Advantages and disadvantages of methods used to limit or prevent expression of a given protein or ncRNA

	CRISPRi	CRISPR ko	shRNAi
Advantages	• Low risk of off-target effects • Potentially inducible and reversible effect • Possibility of non-coding RNA (ncRNA) silencing	• Low risk of off-target effects • Complete knockout • Challenging silencing/removal of ncRNA genes	• High risk of off-target effects • Potentially inducible and reversible effect • Limited potential of ncRNA silencing
Disadvantages	• Residual expression • Selection of efficient sgRNA may require laborious verification	• Requires laborious and time-consuming clonal selection	• Residual expression • Selection of efficient sgRNA requires verification • May evoke interferon response • Potential overload of miRNA biogenesis pathway

## Data Availability

Raw data are available from the corresponding author (JB) upon request. The maps and sequences of vectors are available via Addgene repository.
